# Direct Infection of B Cells by Dengue Virus Modulates B Cell Responses in a Cambodian Pediatric Cohort

**DOI:** 10.3389/fimmu.2020.594813

**Published:** 2021-02-12

**Authors:** Vinit Upasani, Hoa Thi My Vo, Heidi Auerswald, Denis Laurent, Sothy Heng, Veasna Duong, Izabela A. Rodenhuis-Zybert, Philippe Dussart, Tineke Cantaert

**Affiliations:** ^1^ Immunology Unit, Institut Pasteur du Cambodge, Institut Pasteur International Network, Phnom Penh, Cambodia; ^2^ Department of Medical Microbiology and Infection Prevention, University of Groningen and University Medical Center Groningen, Groningen, Netherlands; ^3^ Virology Unit, Institut Pasteur du Cambodge, Institut Pasteur International Network, Phnom Penh, Cambodia; ^4^ Kantha Bopha Children Hospital, Phnom Penh, Cambodia

**Keywords:** infectious diseases, B cell response, dengue viral infection, plasma cell development, DENV entry mechanism

## Abstract

Dengue is an acute viral disease caused by dengue virus (DENV), which is transmitted by *Aedes* mosquitoes. Symptoms of DENV infection range from inapparent to severe and can be life-threatening. DENV replicates in primary immune cells such as dendritic cells and macrophages, which contribute to the dissemination of the virus. Susceptibility of other immune cells such as B cells to direct infection by DENV and their subsequent response to infection is not well defined. In a cohort of 60 Cambodian children, we showed that B cells are susceptible to DENV infection. Moreover, we show that B cells can support viral replication of laboratory adapted and patient-derived DENV strains. B cells were permissive to DENV infection albeit low titers of infectious virions were released in cell supernatants CD300a, a phosphatidylserine receptor, was identified as a potential attachment factor or receptor for entry of DENV into B cells. In spite of expressing Fc*γ*-receptors, antibody-mediated enhancement of DENV infection was not observed in B cells in an *in vitro* model. Direct infection by DENV induced proliferation of B cells in dengue patients *in vivo* and plasmablast/plasma cell formation *in vitro*. To summarize, our results show that B cells are susceptible to direct infection by DENV *via* CD300a and the subsequent B cell responses could contribute to dengue pathogenesis.

## Introduction

Dengue is an arthropod-borne viral disease caused by dengue virus (DENV), a positive sense single-stranded RNA virus belonging to the *Flaviviridae* family and is transmitted by *Aedes* mosquitoes (1). DENV strains are classified into four antigenically distinct serotypes, DENV-1 to -4 (2). Dengue is a major threat to global health, estimated to infect around 390 million people annually affecting more than 100 countries. Around 25% of infections result in clinical disease (3). Dengue disease ranges from mild dengue fever (DF), which is self-limiting, to more severe forms of disease such as dengue hemorrhagic fever (DHF) and dengue shock syndrome (DSS) (4). Previous studies have shown that the more severe forms of dengue occur mainly after secondary infection with a different serotype, leading to skewed and enhanced memory immune responses (5). In humans, cells belonging to the myeloid lineage such as immature and mature dendritic cells, monocytes and macrophages have been shown to be susceptible and permissive to direct DENV infection *in vitro* (6–10). Moreover, these cells can also be infected by a process termed as antibody dependent enhancement (ADE), whereby antibodies produced during previous DENV infection mediate the uptake of DENV *via* Fc receptors (11, 12).

Upon entering the cell, DENV RNA is translated into a single polyprotein which is then cleaved into individual proteins by NS2B3 protease, yielding three structural and seven non-structural (NS) proteins. NS3, one of the non-structural proteins, has helicase and triphosphatase activity which is important for viral replication and is present at the replication sites near the endoplasmic reticulum (13–15). Hence, NS3 protein is only detected in cells upon active infection by DENV and translation of viral proteins, and the detection of DENV NS3 intracellularly in infected cells is indicative of viral replication (8).

Infection with dengue virus has major impacts not only on the myeloid compartment in the blood but also on lymphoid cells (16). For example, it has been shown that circulating CD19^+^ cells are increased in dengue patients (17, 18) and that their subset distribution is significantly altered during infection (19). For example, a massive increase in the frequencies of plasmablasts and plasma cells, reaching up to 50% of circulating B cells during the acute phase of infection has been reported (19, 20). Moreover, enhanced B cell activation and plasma cell development have been observed in hospitalized dengue patients compared to asymptomatic infected patients (21). In addition, we and others have also showed altered antibody-independent B cell responses in dengue patients, as measured by cytokine production and upregulation of activation markers after *in vitro* stimulation (19, 22).

However, it is not known whether these changes in subset distribution and altered functions observed during the acute phase of dengue infection are due to direct infection of B cells by DENV or due to bystander mechanisms as a consequence of viral infection. Indeed, B cells might be susceptible to DENV infection (23–28). Moreover, viral RNA and protein have been demonstrated in secondary lymphoid organs and within the germinal center suggesting that infected B cells can aid in the dissemination of the virus (29–32). B cells also express Fc receptors such as Fc*γ*RIIB and LILRB1 which are implicated in ADE and thus could be targets of enhanced viral infection.

Hence, in this study, we sought to investigate whether B cells were susceptible and permissive to DENV infection both *ex vivo* and *in vitro* and to determine if direct infection altered B cell responses and contributed to viral spread. We observed that B cells from dengue patients were found to support viral replication of laboratory adapted and patient-derived DENV strains both *ex vivo* and *in vitro*. Next, we identified CD300a, a phosphatidylserine receptor, as a potential attachment factor or receptor for entry of DENV into B cells. Infection with DENV induced proliferation of B cells in dengue patients *in vivo* as well as plasmablast and plasma cell formation *in vitro*. Overall, our results show that B cells are susceptible to direct infection by DENV of B cells through CD300a, and the responses of B cells to the infection could play a role in pathogenesis of dengue.

## Materials and Methods

### Ethics Statement

Ethical approval for the study was obtained from the National Ethics Committee of Health Research of Cambodia. Written informed consent was obtained from all participants or the guardians of participants before inclusion in the study.

### Healthy Donor and Patient Recruitment

Venous blood was collected from clinically healthy adult volunteers who presented at the International Vaccination Centre, Institut Pasteur Cambodia. Blood samples were obtained from hospitalized children (≥2 years) who presented with dengue-like symptoms at the Kanta Bopha Hospital in Phnom Penh, Cambodia. The time point for collection of blood samples was within 96 h of fever onset at hospital admittance. Dengue infection was confirmed by diagnostic testing as described below and dengue-negative patients were categorized as febrile controls. Dengue-positive patients were classified according to the WHO 1997 criteria upon hospital discharge into (dengue fever, DF) dengue hemorrhagic fever (DHF) or dengue shock syndrome (DSS). A total of 60 dengue-positive patients were included in the study. In addition, age- and sex-matched healthy donors were recruited from a cluster-based investigation in Kampong Cham province (n = 16) and included for the functional analysis (**Tables 1** and **2**).

**Table 1:** Demographic data of included healthy donors, DENV-negative febrile controls and dengue patients.

	Healthy donors	Febrile controls	dengue patients
**Number of samples**	16	16	60
**M/F ratio**	1.66	0.78	1.14
**Age (mean ± SD)**	9.06 ± 3.78	8.04 ± 4.23	8.6 ± 3.8

**Table 2:** Demographic data and clinical parameters of included dengue patients.

	DF	DHF/DSS
**Number of samples**	48	12
**M/F ratio**	1.09	1.4
**Age (mean** ± **SD)**	8.3 ± 3.8	10.1 ± 3.9
**Day of fever at inclusion (mean, range)**	3.4 (1–4)	3.5 (1–4)
**NS1 + RDT**	34	10
**DENV RT-qPCR +**	47	11
**Viral load (RNA copies/ml) (median, IQR)**	3,480 (7.6–16,300,000)	21.30 (4.4–19,600)
**DENV-1**	26	3
**DENV-2**	19	6
**DENV-3**	0	0
**DENV-4**	2	1
**Secondary infection (%)**	77.1%	83.3%

Patients are characterized according to the WHO 1997 criteria. DENV serotype and viral load were determined by RT-qPCR using a standard curve. Viral load is expressed as RNA copies/ml. NS1 positivity was determined by rapid test. Primary or secondary infection was determined based on HIA results on acute and convalescent samples; IQR, interquartile range; NS1, non-structural protein 1; RDT, rapid diagnostic test; DF, dengue fever; DHF, dengue hemorrhagic fever; DSS, dengue shock syndrome.

### Laboratory Diagnosis

Plasma specimens from patient samples were tested for the presence of DENV using nested RT-qPCR at the Institut Pasteur in Cambodia, the National Reference Center for arboviral diseases in Cambodia (33). Detection of DENV NS1 and anti-DENV IgM/IgG in patient plasma was done using rapid diagnostic tests (SD Bioline Dengue Duo kits, Standard Diagnostics, Abbott, USA). Additionally, anti-DENV IgM was measured with an in-house IgM-capture ELISA (MAC-ELISA), as previously described (34).

### Virus Production

Infections with DENV were carried out using two laboratory/cell culture-adapted reference strains: DENV-1 Hawaii (GenBank: AF425619) and DENV-2 New Guinea C (GenBank: AF038403) and two DENV strains, DENV-1 isolate 91212506 and DENV-2 isolate B0623518, both obtained from Cambodian patients by isolation and passaging two to three times in C6/36 cells. Briefly, *Aedes albopictus* C6/36 cells were infected with virus at an MOI of 0.1 and cultured at 28°C for 5–7 days in Leibovitz 15 medium (Sigma-Aldrich, MO, USA) supplemented with 2% FBS (Gibco, MT, USA), 1% L glutamine (Gibco), 10% tryptose-phosphate (Gibco) and 100 U/ml penicillin–streptomycin (Gibco). DENV was harvested from supernatants of infected C6/36 cells and concentrated using 40% polyethylene glycol (PEG)8000 (Sigma-Aldrich) as previously described (35). The concentrated virus was resuspended in RPMI supplemented with 10% FBS and stored at −80°C. Virus inactivation was done by incubation of virus aliquots under UVS-28 UV Lamp (Analytik Jena, Germany) for 30 min.

### Focus-Forming Assay

Viral titer of produced viral stocks and the permissivity of B cells to DENV infection were determined by focus-forming assay to detect infectious DENV particles in supernatants from infected cells. Briefly, Vero cells (ATCC CCL-81) seeded in 96-well plates were incubated with serially-diluted supernatants from DENV infected B cells and monocytes for 1 h at 37°C and overlaid with Dulbecco’s modified Eagle medium (DMEM; Sigma-Aldrich) supplemented with 3% FBS and 1.8% w/v carboxymethylcellulose (CMC) (Sigma-Aldrich). After 2–3 days, cells were fixed, permeabilized, and stained with DENV serotype-specific polyclonal mouse hyperimmune ascite fluids (Institut Pasteur in Cambodia) as described in Auerswald et al. (36). The number of foci was counted for each dilution, and viral titers were expressed as focus forming units (ffu)/ml.

### Isolation and Infection of B Cells and Monocytes *In Vitro*

PBMCs were isolated from healthy donors using Ficoll-Histopaque density gradient centrifugation. Purified CD19^+^ B cells were isolated from PBMCs by two rounds of separation using positive selection CD19 Microbeads (Miltenyi-Biotec, Germany) as per the manufacturer’s protocol. The purity of B cells obtained was 90–95% as determined by flow cytometry. CD14^+^ monocytes were isolated similarly using CD14 Microbeads (Miltenyi-Biotec, Germany).

For infection experiments, 8 × 10^4^ B cells or monocytes were plated per well in a 96-well plate and infected with DENV-1 or -2 at an MOI of 20 for 90 min at 37°C, 5% CO_2_. The virus inoculum was removed after centrifugation at 1,500pm for 10 min, and cells were washed twice with plain RPMI. The cells were then resuspended in RPMI supplemented with 10% FBS and incubated at 37°C and 5% CO_2_ for 24 h.

### Flow Cytometry

To detect DENV infection in B cells and monocytes from dengue patients and healthy donors infected *in vitro*, cells were stained first with Zombie Aqua Fixable Viability Kit (BioLegend, CA, USA) for live/dead cell gating followed by surface staining with CD19-APC/Cy7 (clone HIB19), CD20 PerCP-Cy5.5 (clone 2H7) or CD14-APC (clone 63D3) (all from BioLegend) for 30 min at 4°C followed by fixation and permeabilization with True-Nuclear Transcription Factor Buffer Kit (BioLegend, USA) as per manufacturer’s protocol. Intracellular staining for detection of DENV infection was done using a rabbit polyclonal anti-DENV NS3 antibody or (GTX124252; GeneTex, CA, USA) or rabbit polyclonal isotype control followed by a goat anti-rabbit secondary antibody conjugated with AF488 (Molecular probes, OR, USA) or anti-DENV E protein (clone 4G2) labelled with AF488 (Molecular probes, OR, USA). Samples were run on BD FACS Canto II (BD Biosciences, NJ, USA) and analyzed by FlowJ0 v10 (BD Biosciences, USA). For the detection of the cytokine BAFF (B-cell activating factor) in the plasma of healthy donors and dengue patients, a LEGENDplex Human B cell Activator Panel immunoassay (BioLegend, USA) was used as per the manufacturer’s instructions. Samples were acquired using BD FACS Canto II and analyzed using LEGENDplex v7.0 (Vigene Tech, MA, USA) software.

### Real-Time PCR on Infected Cells and Cell-Free Supernatants

RNA was extracted from DENV-infected B cells and monocytes from healthy donors and dengue patients using RNeasy Micro Kit (QIAGEN, Germany) as per manufacturer’s protocol. From the supernatants of infected B cells and monocytes, RNA isolation was done using QIAamp Viral RNA Mini kit (QIAGEN, Germany). cDNA was synthetized from extracted RNA with SuperScript II Reverse Transcriptase kit (ThermoFisher, MA, USA) and N6 random primers (Promega, WI, USA) respectively. Real-time PCR was done using primers and probes specific for DENV-1, DENV-2 and DENV-4 (**Table 3**). HPRT (Hypoxanthine-guanine phosphoribosyltransferase) was used as housekeeping gene and 2^−ΔΔCt^ values were calculated. The running conditions for DENV real-time RT-qPCR were as follows: 50°C for 2 min, 95°C for 10 min, 40 cycles of 95°C for 15 s and 60°C for 1 min.

**Table 3:** List of primers and probes used for RT-qPCR.

Serotype	Primer/probe sequence (5′–3′)
DENV-1 fw	ATCCATGCCCAYCACCAAT
DENV-1 rev	TGTGGGTTTTGTCCTCCATC
DENV-1 Probe	FAM-TCAGTGTGGAATAGGGTTTGGATAGAGGAA-BHQ1
DENV-2 fw	TCCATACACGCCAAACATGAA
DENV-2 rev	GGGATTTCCTCCCATGATTCC
DENV-2 Probe	FAM-AGGGTGTGGATTCGAGAAAACCCATGG-BHQ1
DENV-4 fw	GYGTGGTGAAGCCYCTRGAT
DENV-4 rev	AGTGARCGGCCATCCTTCAT
DENV-4 Probe	Cyan500-ACTTCCCTCCTCTTYTTGAACGACATGGGA-BHQ1
HPRT fw	TGACACTGGCAAAACAATGCA
HPRT rev	GGTCCTTTTCACCAGCAAGCT
HPRT Probe	FAM-CTTGACCATCTTTGGATTATACTGCCTGACCA-BHQ1

### Identification of Receptor on B Cells for Dengue Virus

CD19^+^ B cells were incubated with different concentrations (1–10 ug/ml) of an IgG2a monoclonal antibody directed against CD300a (clone P192; LSBio, WA, USA) for 30 min, washed, and infected with DENV-2 at an MOI of 20 for 24 h. An isotype-matched monoclonal antibody was used as a negative control. Percentages of DENV-infected B cells were determined by flow cytometry using anti-DENV NS3 antibody as described above, and fold change in percentage of infected cells with respect to the control was calculated.

### Antibody-Dependent Enhancement Assay

Human monoclonal antibody G10 (kind gift from Katja Fink, A*STAR, Singapore) is specific for the fusion loop of DENV E protein and has been shown to mediate the antibody-dependent enhancement of DENV infection *in vitro* (37). Human myelomonocyte cell line U937 (ATCC CRL-1593.2) was cultured in RPMI (Gibco) supplemented with 10% FBS (Gibco), 100 U/ml penicillin–streptomycin (Gibco), and 1% L glutamine (Gibco) (38). Serum was obtained from a pediatric patient with primary DENV-2 infection at day 8 after onset of symptoms (early convalescent phase). The G10 antibody and DENV-2 patient serum were serially diluted five-fold (1:100 to 1:1,562,500) in RPMI and incubated with DENV-1 virus corresponding to MOI of 1 for 1 h at 37°C, 5% CO_2_. Immune complexes were then transferred to purified B cells from healthy donors and incubated for 90 min at 37°C, 5% CO_2_. Direct infection with DENV in the absence of G10 antibody was used as control. After infection, cells were washed and incubated for 72 h at 37°C, 5% CO_2_. Cells were surface stained with Zombie Aqua (AmCyan) viability dye (BioLegend) for live/dead cell gating and then fixed, permeabilized, and stained for the presence of DENV using anti-DENV E protein antibody (clone 4G2, ATCC HB-112) labeled with AF488 (Molecular probes). Fold change of infection was calculated for each serum dilution with respect to direct DENV infection to represent enhancement of DENV infection.

### *In Vitro* Plasma Cell Differentiation

Purified CD19^+^ B cells from healthy donors**’** PBMCs were infected with DENV-1 at MOI of 5 for 90 min, washed and cultured in the presence of CD40L (0.25 μg/ml; ITS Vietnam), IL-2 (1 ng/ml; Peprotech, NJ, USA), and IL-21 (50 ng/ml; Peprotech) for 6 days. The cells were harvested and stained with Zombie Aqua (AmCyan) viability dye (BioLegend) for live/dead cell gating followed by antibodies CD19 PE/Cy7 (clone HIB19), CD20 PerCp/Cy5.5 (clone 2H7), CD27 APC/Cy7 (clone O323), CD38 APC (clone HB7) and CD138 BV421 (clone MI15), and the percentages of CD27^+^CD38^+^ plasmablasts and CD27^+^CD138^+^ plasma cells were determined in uninfected and infected B cells with or without stimulation.

### B Cell Proliferation Assay

B cells isolated from healthy donors were labeled with carboxyfluorescein diacetate succinimidyl ester (CFSE) (Biolegend) and either infected with DENV-2 at an MOI of 5 or remained uninfected. After 2 h of inoculation, cells were washed to remove the inoculum and stimulated with CpG oligodeoxynucleotides (1 μg/ml; Invivogen, San Diego, CA, USA) and F(ab′)_2_ anti-IgM antibody (4 μg/ml; Jackson ImmunoResearch, PA, USA) or remained unstimulated. Cells were cultured in RPMI supplemented with 10% FBS for 6 days. The cells were then harvested and stained with Zombie Aqua (AmCyan) viability dye (BioLegend) followed by CD19 PE/Cy7 (clone HIB19) and CD20 PerCp/Cy5.5 (clone 2H7) to identify live B cells and analyzed for the expression of CFSE in the FITC channel. Proliferation was measured as the percentage of B cells with decreased intensity of CFSE compared to unstimulated B cells.

### Statistical Analysis

Statistical analyses were done using GraphPad Prism 7.00 software (GraphPad Software, Inc., La Jolla, CA, USA). Since the data did not pass the criteria for normality using D’Agostino & Pearson normality test, the non-parametric Mann–Whitney *U-*test was used to compare data between two groups or by non-parametric paired Wilcoxon matched pairs signed rank test for paired data. Statistical analysis of data with more than two groups was done using the Kruskal–Wallis test followed by Dunn’s post-test for multiple comparisons. For comparing paired samples between three conditions, Friedman’s test was used. Correlations were calculated by Spearman analysis. For all analyses, p <0.05 was considered significant.

## Results

### B Cells Are Susceptible to Dengue Virus Infection *In Vivo*

Previously published studies on susceptibility of immune cells to DENV infection have primarily used the 4G2 antibody, a pan-flaviviral antibody binding to the fusion loop of the Envelop (E) protein. However, the presence of E protein cannot distinguish between binding/internalization and productive infection. Therefore, we aimed to detect viral non-structural protein 3 (NS3). NS3 protein is only detected in cells upon active infection by DENV, and translation of viral proteins and the intracellular detection of DENV NS3 is indicative of viral replication (16, 39). To validate the anti-NS3 antibody, we infected C6/36 cells, which are highly susceptible to DENV infection, with DENV-1 at MOI of 5 for 24 h and stained with anti-DENV NS3 antibody and its corresponding isotype control. Representative histogram of NS3 staining in C6/36 cells is shown in **Supplementary Figure 1A**. To further confirm the specificity of the anti-DENV NS3 antibody, C6/36 mosquito cell line and primary monocytes, well known targets of DENV infection, were infected with DENV and UV-inactivated DENV particles (UV-DENV). An increase in percentage of NS3^+^ cells was observed in DENV infected cells compared to uninfected and UV-DENV infected cells (**Supplementary Figure 1B**).

**Figure 1:**
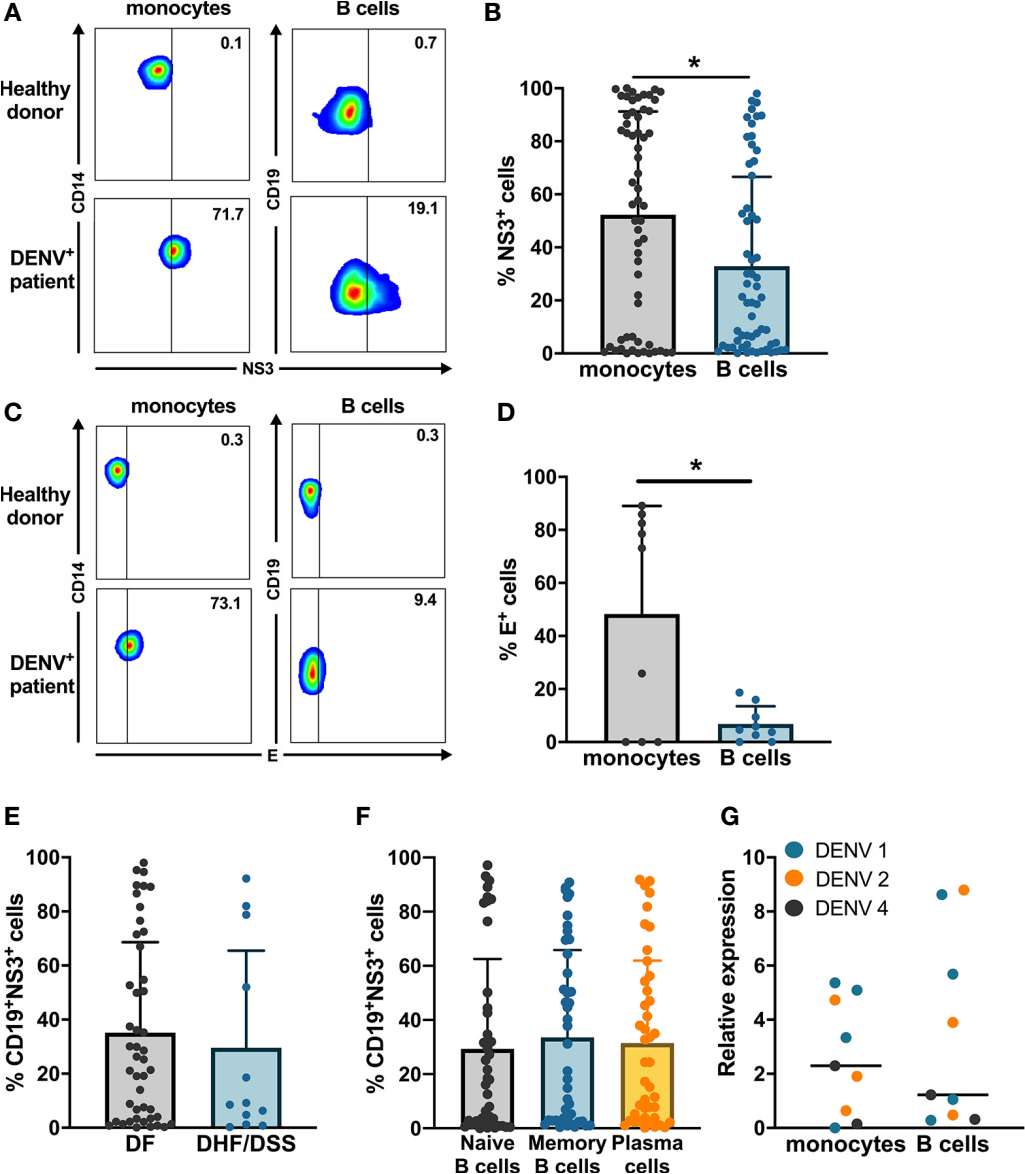
*Ex vivo* detection of DENV in B cells from dengue patients. PBMCs from patients in the acute phase of DENV infection (n = 60) were stained on the surface with antibodies for immune cell markers and intracellularly with anti-DENV NS3 antibody or pan flaviviral fusion loop specific 4G2 antibody. **(A, B)** Representative plots for NS3 staining in CD14^+^ monocytes and CD19^+^ B cells. The percentage of NS3^+^ cells were determined for CD14^+^ monocytes and CD19^+^ B cells. **(C, D)** Representative plot for anti-E staining in CD14^+^ monocytes and CD19^+^ B cells. Percentages of E^+^ monocytes and B cells were determined in a subset of dengue patients (n = 9). **(E)** DENV patients were classified as DF (n = 46) and DHF/DSS (n = 12) as per WHO 1997 classification, and the percentage of CD19^+^NS3^+^ cells was determined. p-values were calculated using Mann–Whitney U test for comparing two groups. **(F)** B cells from dengue patients were gated for naive B cells (CD19^+^CD27^−^), memory B cells (CD19^+^CD27^+^CD138^−^) and plasma cells (CD19^+^CD27^+^CD138^+^), and the percentage of NS3^+^ cells was determined. **(G)** CD14^+^ monocytes and CD19^+^ B cells were isolated from PBMCs from dengue patients by magnetic sorting. RT-qPCR was done for DENV by serotype-specific PCR and HPRT. Relative expression was calculated using 2^−ΔΔCt^ method. For all panels, P-values were calculated using Mann–Whitney U test for comparing two groups. Bars and lines represent mean and standard deviation (SD). (*P < 0.05).

In order to investigate whether B cells are susceptible to DENV infection *in vivo*, we obtained PBMCs from a cohort of 60 acute-infected Cambodian children with RT-qPCR confirmed DENV infection and stained with anti-DENV NS3 and E antibodies (**Figures 1A–D**, **Supplementary Figure 2**). Only a subset of patients was stained with anti-E, when sufficient PBMC could be purified due to the low amount of blood obtained from the pediatric cases.

The percentage of E^+^ cells was significantly higher in CD14^+^ monocytes compared to CD19^+^ B cells (**Figures 1C, D**). We confirmed productive infection in both CD14^+^ monocytes and CD19^+^ B cells as we could detect DENV NS3 protein. In parallel, the percentage of CD19^+^NS3^+^ B cells in dengue patients was significantly lower compared to CD14^+^NS3^+^ monocytes (20.9 *vs* 56.5%; p < 0.05) (**Figures 1A, B**). When the patients were stratified according to disease severity, no difference was observed in the percentages of CD19^+^NS3^+^ B cells between patients with DF (n = 46) and those with DHF/DSS (n = 12) (**Figure 1E**). Since different subsets of B cells have different functions, we wanted to determine which subset of B cells is particularly susceptible to DENV infection. Therefore, using flow cytometry we classified CD19^+^ B cells as naïve B cells (CD19^+^CD27^−^), memory B cells (CD19^+^CD27^+^CD138^−^) and plasma cells (CD19^+^ CD27^+^CD138^+^) (**Supplementary Figure 3**). The percentage of DENV NS3^+^ cells was similar between naïve, memory B cells, and plasma cells suggesting that all B cell subsets seem to be equally susceptible to DENV infection (**Figure 1F**). As we observed wide variability in the percentages of infected cells, we aimed to see if this correlated to biological parameters of disease severity. However, no correlations could be observed between percentages of NS3^+^ infected B cell subsets and hematocrit or platelet counts (**Supplementary Figure 2**). To confirm the presence of DENV RNA, we performed RT-qPCR with DENV-serotype specific primers on purified CD19^+^ B cells and CD14^+^ monocyte fractions isolated from patients (n = 9) Relative expression of DENV (2^−ΔΔCt^) was calculated using HPRT as a reference housekeeping gene. Hence, we measure presence of viral RNA which can originate both from surface bound and internalized viral particles. In parallel to the detection of DENV-NS3 protein, the relative expression of DENV was higher in CD14^+^ monocytes compared to CD19^+^ B cells even though the difference was not significant possibly due to the small sample size (**Figure 1G**).

### B Cells Are Susceptible and Permissive to Dengue Virus Infection *In Vitro*

**Figure 2:**
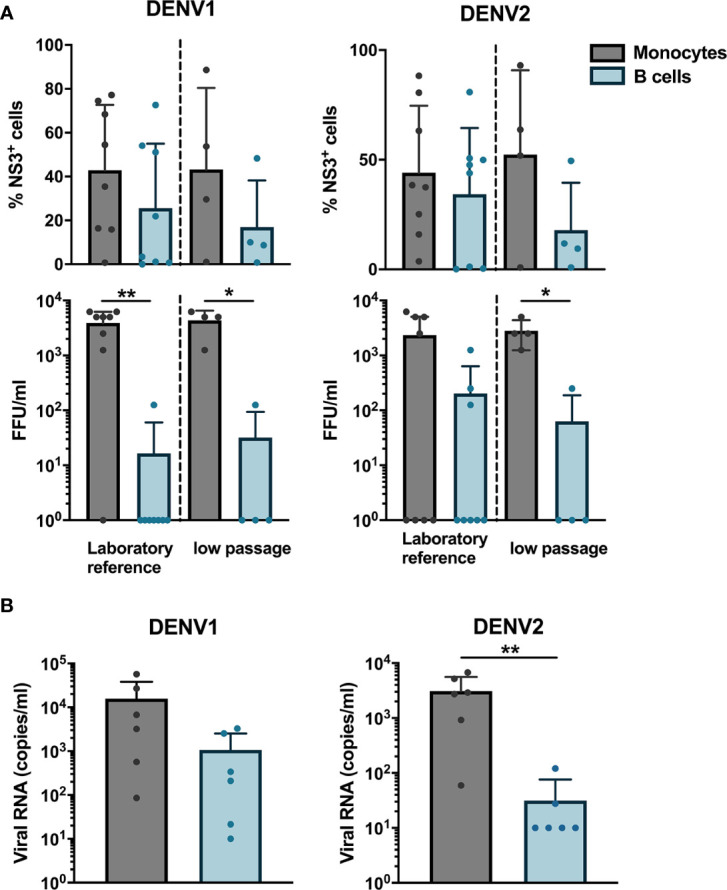
Susceptibility and permissiveness of B cells to DENV infection *in vitro*. **(A)** CD14^+^ monocytes and CD19^+^ B cells were isolated from PBMCs from healthy donors by magnetic sorting and infected with reference or low-passaged DENV-1 and DENV-2 strains at MOI of 20. At 24 h post infection, the cells were stained intracellularly with anti-DENV NS3 antibody. Infectious viral progeny in supernatants from healthy donors (n = 6) infected with DENV-1 and -2 reference or low-passaged stains at MOI 20 for 24 h was determined by focus-forming assay. **(B)** Total DENV viral RNA copies in supernatants from healthy donors (n = 6) infected with DENV-1 and -2 reference stains at MOI 20 for 24 h was determined by RT-qPCR. For all panels, bars and lines represent mean and standard deviation (SD). (*P < 0.05; **P < 0.01).

Next, we wanted to determine if B cells isolated from healthy donors were susceptible to DENV infection *in vitro*. CD19^+^ B cells and CD14^+^ monocytes isolated from healthy donors were infected with either of a laboratory reference strains DENV1 Hawaii, DENV2 New Guinea C or low passaged DENV-1 and -2 isolated from acute dengue patients. The percentages of DENV NS3^+^ cells were similar between monocytes and B cells infected with the low-passaged DENV-1 or DENV-2. Of interest, no difference was observed between percentages of NS3^+^ cells in B cells and monocytes infected with laboratory reference strains and low-passaged DENV-1 and -2 strains (**Figure 2A**).

As we observed that B cells are susceptible to DENV infection, we investigated whether B cells are permissive to DENV, *i.e.*, the ability of the virus to complete its replication cycle in B cells and release complete, mature virions which can infect new cells. To answer this question, a focus forming assay was performed on Vero cells using supernatants from B cells and monocytes infected *in vitro* with DENV. Monocytes produced higher titers of both DENV-1 and DENV-2, except for one donor where no foci were observed. A low virus titer could be observed in cells incubated with supernatants from DENV-infected B cells from one donor infected with DENV-1 reference strain and from three donors infected with DENV-2 reference strain (**Figure 2A**). A similar trend was observed for monocytes and B cells infected with low passaged DENV-1 and -2 (**Figure 2A**). Furthermore, to estimate the total amount of DENV particles, we measured RNA copies in the supernatants from monocytes and B cells from six healthy donors infected with laboratory reference DENV-1 and -2. Higher quantities of viral RNA copies were detected in supernatants from DENV-infected monocytes compared to B cells, especially for DENV-2 where the differences were significant (p < 0.01) (**Figure 2B**). Interestingly, detectable quantities of RNA copies were observed in B cells from five out of six donors infected with DENV-1 compared to 2 for DENV-2. Taken together, these results indicate that DENV can productively infect B cells; however, this seems to be donor-dependent and with low efficiency.

### Identification of Entry Mechanism for Dengue Virus in B Cells

**Figure 3:**
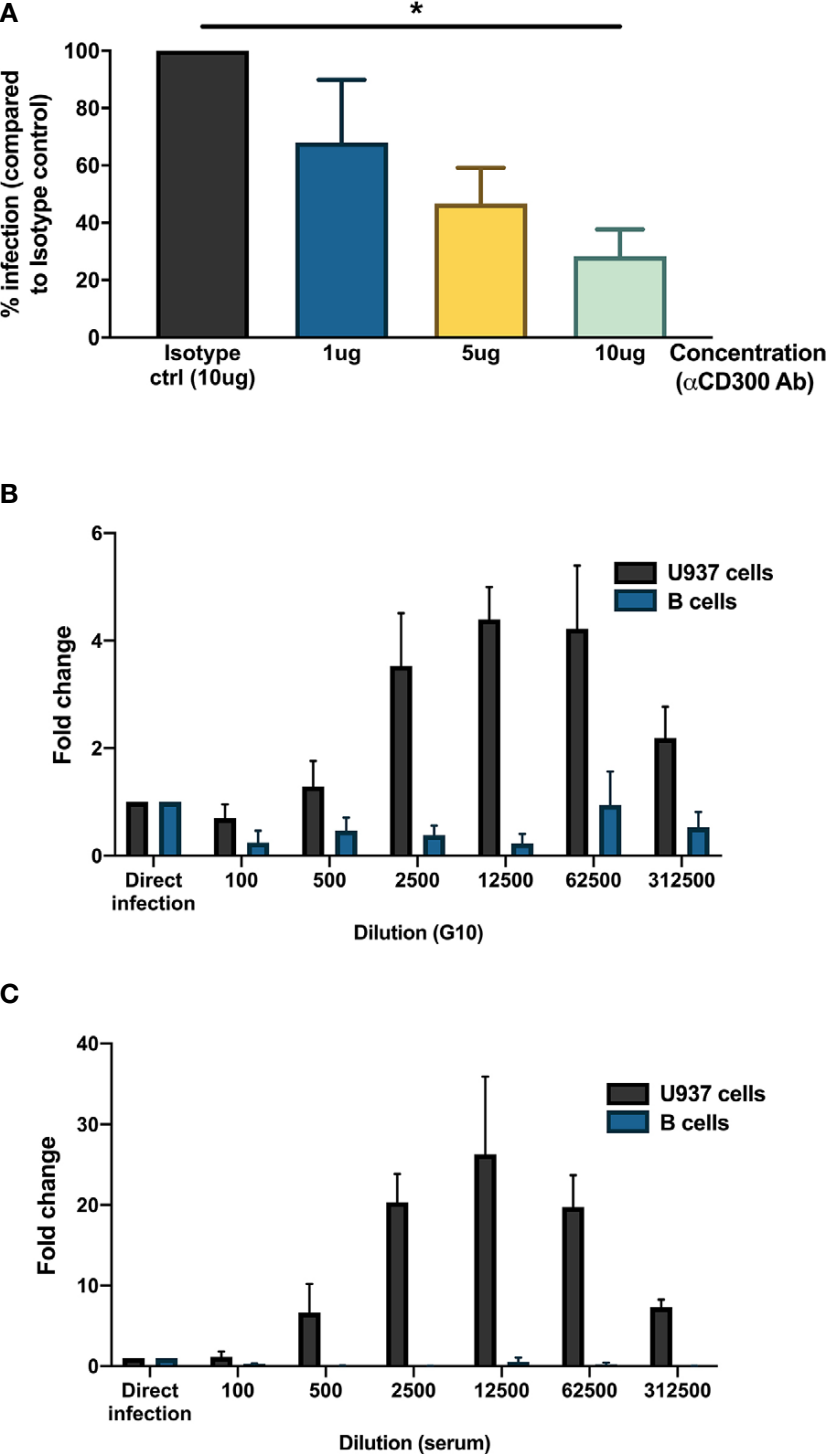
Mechanisms for DENV entry into B cells. **(A)** CD19^+^ B cells were isolated from PBMCs from healthy donors (n = 4) by magnetic sorting, incubated with different concentrations of blocking antibody against CD300a or isotype control antibody and infected with reference DENV-1 or DENV-2 strains at MOI 20. At 24 h post infection, cells were stained with anti-DENV NS3 antibody. Percentage of infection is represented with respect to isotype control. **(B, C)** Monoclonal antibody G10 or serum from patient with DENV-2 infection was serially diluted five-fold (1:100 to 1:1,562,500) in RPMI and incubated with DENV-1 virions corresponding to MOI of 1 for 1 h at 37°C, 5% CO_2_. These immune complexes were then transferred to U937 cells and B cells and incubated for 90 min at 37°C, 5% CO_2_. Direct infection with DENV in the absence of G10 antibody or patient serum was used as control. At 72 h post infection, cells were fixed, permeabilized, and stained with anti-E antibody (clone 4G2). Fold change is represented for each dilution with respect to direct infection. Bars represent mean and SEM, where the experiment was replicated with three different healthy donors. (*P < 0.05).

As we observed the presence of DENV antigen in B cells upon infection *in vitro*, we aimed to identify a potential attachment factor or receptor for entry of DENV into B cells. Based on published studies, we identified CD300a, belonging to the CD300 family of phospholipid receptors, as a potential receptor involved in binding or entry of DENV (40). CD300a is moderately expressed on all B cell subsets and has been shown to be downregulated during HIV infection (41, 42). Higher expression of CD300a is observed in memory B cells and plasmablasts compared to naïve B cells (42). To test whether CD300a could play a role in DENV infection in B cells, CD19^+^ B cells were infected with DENV-1 in the presence of blocking antibody for CD300a or isotype control. Blocking of CD300a leads to a decrease in percentage of DENV-infected CD19^+^NS3^+^ cells in a concentration-dependent manner but had no effect on viability of CD19^+^ B cells. A significant decrease in infection of more than 50% was observed between direct infection and 10 ug concentration of CD300a blocking antibody (p < 0.05) (**Figure 3A**). However, complete abrogation of DENV infection in B cells was not observed even at higher concentrations of CD300a blocking antibody suggesting possible involvement of other receptors or attachment factors in entry of DENV into B cells.

Since B cells express Fc*γ* receptor Fc*γ*RIIB, we wanted to test whether B cells are susceptible to antibody-mediated DENV infection. Therefore, DENV-1 at MOI of 1 was incubated with serial dilutions of a monoclonal antibody (clone G10) with antibody-dependent enhancement potential (37) or patient serum obtained 8 days after a primary DENV-2 infection and tested on B cells obtained from healthy donors (n = 3) and Fc*γ*RIIA bearing cells U937 as positive control. Here, whereas both the monoclonal antibody G10 and patient serum induced ADE in U937 cells, no enhancement of infection was observed with the primary B cells isolated from healthy donors. (**Figures 3B, C** **)**. This indicates that antibody-dependent enhancement of DENV infection does not occur in B cells.

### Infected B Cells Show a Higher Proliferation History

**Figure 4:**
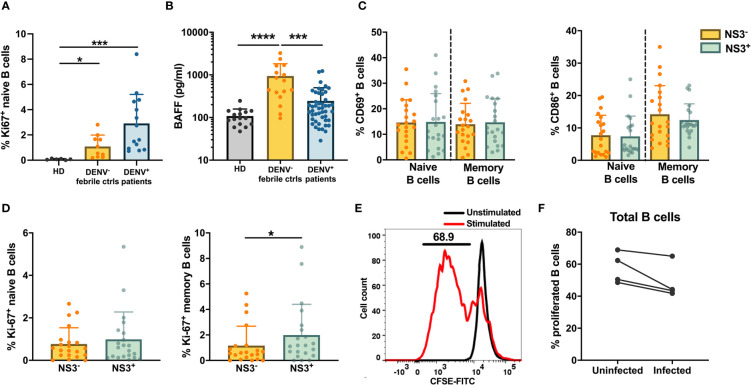
Activation and proliferation of DENV-infected B cells *in vivo*. **(A)** Frequencies of Ki-67^+^ naive (CD19^+^CD27^−^) B cells in healthy donors, DENV-negative febrile controls and dengue patients. **(B)** Concentrations of B-cell activating factor (BAFF) were analyzed in plasma of age-matched healthy donors, febrile controls and and patients with acute dengue infection. **(C, D)** CD19^+^ B cells from dengue patients were stained with B cell subset markers and anti-DENV NS3 antibody. Frequencies of **(C)** activated CD69^+^ and CD86^+^ and **(D)** proliferating Ki-67^+^ B cells within NS3^−^ and NS3^+^ populations of naive (CD19^+^CD27^-^) and memory B cells (CD19^+^CD27^+^CD138^−^) from dengue patients. **(E)** Representative histogram to determining percentage of proliferated B cells upon stimulation with F(ab′)_2_ anti-IgM antibody and CpG compared to unstimulated cells. **(F)** Frequencies of proliferated B cells isolated from healthy donors (n = 4) infected with or without DENV-2 reference strain *in vitro* followed by stimulation with F(ab′)_2_ anti-IgM antibody and CpG for 6 days. For all panels, P-values were calculated using Mann–Whitney U test for comparing two groups. Bars and lines represent mean and standard deviation (SD). (*P < 0.05; ***P < 0.001; ****P < 0.0001).

Regardless of the observed low permissiveness of B cells to DENV, direct infection could alter B cell responses, such as proliferation. Therefore, we looked at the expression of intracellular proliferation marker Ki-67 in total naïve CD19^+^ B cells from dengue patients, healthy, and febrile controls. Increased expression of Ki-67 in naïve B cells was observed in dengue patients compared to healthy controls and dengue-negative febrile controls (p < 0.05) suggesting that B cells are proliferating more in dengue patients (**Figure 4A**). Hence, we analyzed the serum concentrations of B-cell activating factor (BAFF), a cytokine produced by cells of the myeloid lineage and is known to be a potent activator of B cells in plasma of DENV patients. Indeed, concentrations of BAFF were significantly higher in dengue-infected patients compared to healthy donors, which could contribute to the increased proliferation observed (**Figure 4B**). However, BAFF serum concentrations were even more increased in febrile controls.

Next, we questioned if direct infection of B cells by DENV alters the activation and proliferation of DENV-infected B cells. Therefore, B cells from dengue patients were stained with anti-Ki-67 and anti-CD69/anti-CD86, two activation markers, and with anti-NS3 to identify infected cells. Cells were classified as naïve (CD19^+^CD27^−^) and memory (CD19^+^CD27^+^) B cells and then separated as uninfected (NS3^−^) and infected (NS3^+^) cells (**Supplementary Figure 4**). The presence of DENV seemed to have no effect on the activation of naïve and memory B cells as the percentages of CD69^+^CD86^+^ cells were similar between NS3^−^ and NS3^+^ cells (**Figure 4C**). However, higher proliferation was seen in infected memory B cells compared to uninfected cells (p < 0.05) indicating that DENV-infected B cells show enhanced proliferation (**Figure 4D**). Here, no difference was observed for infected *versus* non-infected naïve B cells. These data suggest that the observed increase in naïve B cell proliferation compared to controls as observed in **Figure 4A** could be due to an indirect effect, rather than direct B cell infection, such as increased BAFF concentrations.

Therefore, we investigated whether infection of B cells by DENV *in vitro* induced their proliferation. For this, B cells from healthy donors (n = 4) were labeled with CFSE and stimulated with CpG and F(ab′)_2_ anti-IgM antibody for 6 days. Proliferation was defined as percentage of B cells with lower CFSE signal (CFSE^low^) upon stimulation compared to unstimulated cells (**Figure 4E**). We evaluated proliferation in B cells that were either infected with DENV-2 or were uninfected at the start of the culture. No difference was observed in the percentage of CFSE^low^ B cells between uninfected and infected B cells of the same individuals (median: 56.4 *vs* 43.8%) (**Figure 4F**). Altogether, these data suggest that the observed increase in proliferation in dengue patients *in vivo* can be attributed to direct DENV infection and bystander mechanisms such as increased serum BAFF concentrations.

### Direct Infection of B Cells by Dengue Virus Increases the Differentiation to Plasmablasts

**Figure 5:**
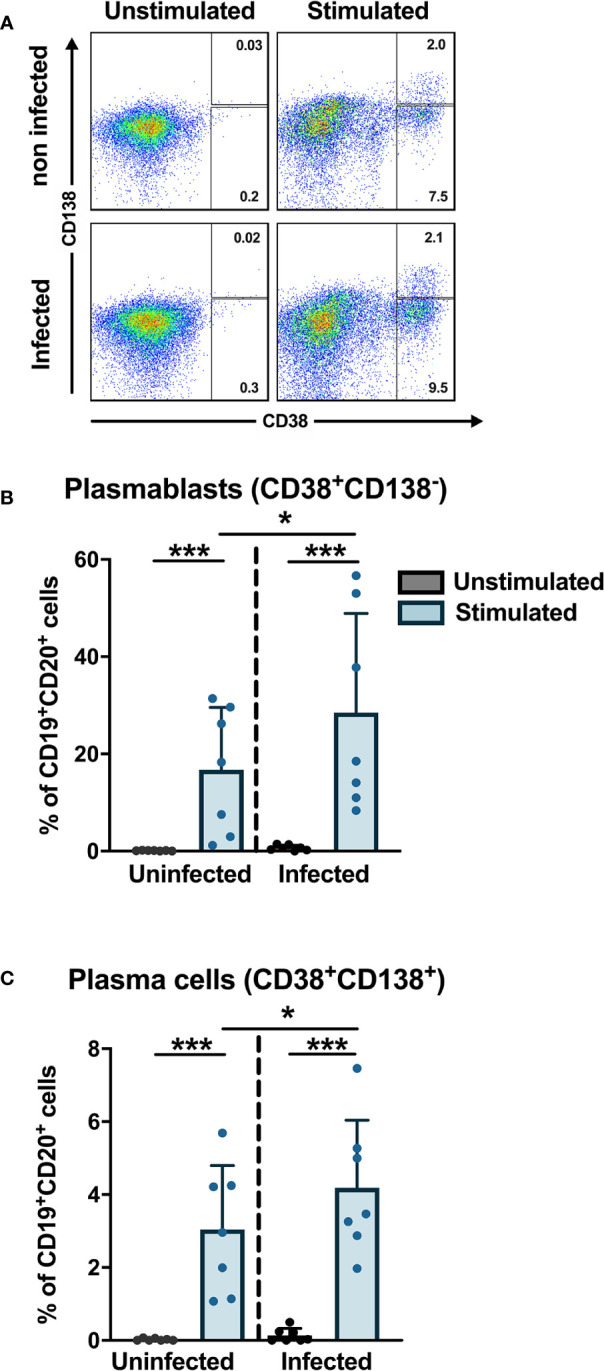
*In vitro* plasmablast and plasma cell development after DENV infection in B cells. B cells isolated from healthy donors (n = 6) were infected with or without DENV-1 reference strain or uninfected and cultured in the presence of IL-2, IL-21, and CD40L for 6 days and stained for B cell subset markers. Percentages of CD38^+^CD138^−^ plasmablasts and CD38^+^CD138^+^ plasma cells were determined within the CD19^+^CD27^+^ B cell population. Bars and lines represent mean and standard deviation (SD). P-values were calculated with Wilcoxon match pairs signed ranked test and Mann–Whitney U test to compare conditions in different groups. (*P < 0.05; ***P < 0.001).

Previous studies have shown that the frequencies of plasmablasts are significantly increased during the early phase of DENV infection where plasmablasts can account for more than 50% of circulating CD19^+^ B cells (19, 20). To check if DENV can induce differentiation of B cells into plasmablasts and plasma cells *in vitro*, purified CD19^+^ B cells from healthy donors were infected with DENV-1 and cultured with or without stimulation with CD40L, IL-2 and IL-21 for 6 days, which mimics the conditions of the germinal center *in vitro*. From the CD19^+^ purified cells, we gated for plasmablasts (CD20^+^CD27^+^CD38^+^CD138^−^) and plasma cells (CD20^+^/^dim^CD27^+^CD38^+^CD138^+^) (**Supplementary Figure 5**), and their percentages were determined As expected, stimulation of B cells with cytokines induced differentiation into plasmablasts and plasma cells. Interestingly, higher frequencies of plasmablasts and plasma cells were observed when stimulation was done after infection compared to uninfected stimulated B cells (median: plamsablasts—16.1 *versus* 18.5% and plasma cells—2.6 *versus* 3.5%; p < 0.05) (**Figure 5**). These data suggest that DENV infection of B cells increases the differentiation to plasmablasts and plasma cells.

## Discussion

The aim of this study was to investigate the susceptibility and permissivity of B cells to infection with DENV and their response to direct infection with the virus. DENV has been shown to infect a variety of cell types such as monocytes, dendritic cells, and T lymphocytes (6, 8, 9, 43). Studies have shown that B cells can be infected by DENV and may contribute to the spread of the virus to the germinal center (23–32). Here, we showed that B cells are susceptible and permissive to DENV infection both *ex vivo*, in patients with acute DENV infection, and *in vitro*.

The presence of NS3 protein in infected cells indicates uncoating and translation of viral RNA which is a pre-requisite for initiating DENV replication (16, 39) Therefore, the detection of NS3 protein is indicative of viral replication. Indeed, we detected NS3^+^ B cells in the blood of dengue patients, suggesting that B cells are susceptible to infection by DENV. Percentages of DENV NS3^+^ and E^+^ B cells were lower compared to monocytes. Moreover, B cells appeared less permissive to infection as infection of CD19^+^ B cells did not always lead to the production of infectious virus. Infection could result in immature or functionally impaired virions that may not be capable of infecting other susceptible cells as seen by the low titer of infectious DENV in supernatants from B cells.

We identified CD300a as potential attachment/entry receptor for DENV in B cells. CD300a belongs to the CD300 family of phospholipid receptors and recognizes phosphatidylserine and phosphatidylethanolamine, which are exposed on the outer side of the plasma membrane of dead and activated cells. In addition, these molecules can be present in viral envelopes derived from the lipid bilayer of the host cell plasma membrane, as is the case for DENV. Indeed, Carnec et al. have described that human and mouse CD300a bind the four DENV serotypes and enhance the infection through clathrin-mediated endocytosis (40). This was also demonstrated for Yellow fever, West Nile, and Chikungunya viruses (40). Furthermore, blocking of CD300a receptor in monocyte-derived macrophages naturally expressing CD300a leads to a decrease in DENV infection (40). In our study, we identified CD300a as an attachment/entry factor for DENV in B cells. We observed that when CD300a was blocked on CD19^+^ B cells, a significant decrease in infection was observed in a concentration-dependent manner suggesting CD300a can be an attachment/entry factor or receptor for DENV in B cells. However, infection was not completely abrogated, probably due to the high MOI used in our experiments. It could also indicate a potential role for other attachment factors or receptors like TIM-1, which needs to be investigated further (44, 45). B cells express Fc*γ* receptor Fc*γ*RIIB and Fc-like receptor LILRB1, both of which are implicated in the mechanism of antibody-dependent enhancement and have shown to be upregulated after DENV infection (19, 46, 47). Hence, theoretically, B cells could be susceptible to antibody-mediated DENV infection. We tested this hypothesis and could not observe an enhancement of infection using a monoclonal anti-E antibody and patient serum, whereas ADE was readily observed with Fc*γ*R bearing U937 cells. Indeed, Fc*γ*RIIB receptor contains an immunoreceptor tyrosine-based inhibitory motif (ITIM) and has been shown to inhibit ADE of DENV infection (48).

We observed increased proliferation as measured by presence of proliferation marker Ki-67 in total B cells from dengue patients compared to febrile controls and in DENV-infected B cells compared to non-infected cells *ex vivo* suggesting that infection with DENV may induce the proliferation of B cells. However, after *in vitro* stimulation of purified B cells *via* BCR/TLR9 and DENV infection, we did not see an increase in proliferation indicating that perhaps contact with other (infected) PBMCs may be needed for proliferation. It remains to be investigated which stimuli account for the observed increased proliferation *in vivo*. One of the cytokines stimulating B cell proliferation is BAFF (B-cell activating factor). The protein exists as a soluble monomer in the serum or as a homotrimer on the surface of myeloid cells and binds to tumor-necrosis factor receptors BAFF-R, BCMA and TACI expressed on B cells, triggering the activation and proliferation of B cells (49). Indeed, we observed increased plasma concentrations of BAFF in dengue patients compared to healthy donors. The source of BAFF in our DENV patient cohort may be CD14^++^CD16^+^ intermediate monocytes as shown in a study by Kwissa et al. in which frequencies of CD14^++^CD16^+^ intermediate monocytes positively correlated with concentrations of BAFF in blood of dengue patients (50).

Here, we investigated the capacity of DENV to induce differentiation of B cells into antibody-secreting plasmablasts and plasma cells *in vitro*. Upon culturing with IL-2, IL-21, and CD40L, we observed increased differentiation of DENV-infected B cells into CD27^+^CD38^+^ plasmablasts and CD138^+^ plasma cells compared to uninfected cells. These findings suggest that DENV infection of B cells increases the differentiation to plasmablasts and plasma cells, independent of the BCR specificity of these cells. This could play a role in disease pathogenesis through antibody-independent functions such as production of plasma cell-derived cytokines IL-35 and IL-10 (51). Alternatively, production of cross-reactive and autoreactive antibodies by plasmablasts could play a role in disease pathogenesis. During DENV infection, several autoantibodies against host factors such as endothelial cells, platelets, and components in coagulation pathways were observed (52–55). Moreover, direct infection could stimulate B cells with BCRs with weak affinity for DENV and/or cross-reactive BCRs to differentiate to plasmablasts and produce IgG with low affinity or little specificity that could contribute to antibody-dependent enhancement (56, 57). Finally, direct infection of B cells by DENV could trigger intracellular responses leading to changes in IgG Fc glycosylation pathways. Indeed, altered abundance of total and DENV-specific IgG afucosylated forms have been observed during acute DENV infection correlating with platelet count and haematocrit (58).

In summary, we have shown that B cells are susceptible to laboratory adapted and low-passaged DENV strains. Infectious virus can be detected after B cell infection, albeit the amount is low. We identified CD300a, a phosphatidylserine receptor, as a potential attachment factor/entry receptor of DENV into B cells. Infection with DENV induced proliferation of B cells in dengue patients *in vivo* and plasmablast/plasma cell formation *in vitro*. The responses of B cells to direct DENV infection could play a role in pathogenesis of DENV.

## Data Availability Statement

The original contributions presented in the study are included in the article/**Supplementary Material**; further inquiries can be directed to the corresponding author.

## Ethics Statement

The studies involving human participants were reviewed and approved by the National Ethics Committee of Health Research of Cambodia. Written informed consent to participate in this study was provided by the participants’ legal guardian/next of kin.

## Author Contributions

TC conceived the project, designed the study, analyzed and interpreted data, and wrote the manuscript. VU conducted the experiments, performed the data analysis, and prepared the manuscript. DL and SH coordinated the recruitment of dengue patients. HV and HA conducted the experiments and performed the data analysis. IR-Z interpreted the data. PD, VD, and TC classified the dengue patients and selected the samples. HV, HA, IR-Z, and PD revised the manuscript. All authors contributed to the article and approved the submitted version.

## Funding

TC was funded by the Institute Pasteur International Network and is an HHMI-Wellcome Trust International Research Scholar (208710/Z/17/Z). VU was funded by the Institute Pasteur International Network Calmette and Yersin Ph.D. scholarship.

## Conflict of Interest

The authors declare that the research was conducted in the absence of any commercial or financial relationships that could be construed as a potential conflict of interest.

## Supplementary Material

Supplementary Figure 1 Optimization of anti-DENV NS3 antibody. **(A)** C6/36 cells were infected with DENV-1 at MOI of 20 for 24 h and stained with rabbit polyclonal anti-DENV NS3 antibody and secondary goat antibody conjugated with AF488. A non-specific rabbit polyclonal antibody was used as negative control. **(B)** C6/36 cells and CD14^+^ monocytes isolated from healthy donors were infected *in vitro* with DENV1 or UV-inactivated DENV1 (UV-DENV1) at MOI 10 and stained at 24 h post infection with rabbit polyclonal anti-DENV NS3 antibody and secondary goat antibody conjugated with AF488.

Supplementary Figure 2 No correlation between biological parameters and percentages of DENV-infected B cells. Correlations between platelet counts and hematocrit levels at hospital admittance and percentages of DENV-infected B cell subsets were determined by Spearman’s correlation.

Supplementary Figure 3 Representative gating strategy used to detect DENV infection in B cells and monocytes in PBMCs from dengue patients. PBMCs were gated for lymphocytes and monocytes followed by removal of doublets. Single CD14^+^ monocytes and CD19^+^ B cells were gated and DENV NS3^+^ cells were selected.

Supplementary Figure 4 Representative gating strategy used to detect DENV infection in B cell subsets in PBMCs from dengue patients. Lymphocytes from PBMCs were further gated for CD19^+^ B cells. Based on expression of CD19 and CD27, total B cells were further gated as naïve B cells (CD19^+^CD27^−^). CD27^+^ B cells were gated as memory B cells (CD19^+^CD27^+^CD138^−^) and antibody secreting cells (CD19^+^CD27^+^CD138^+^) based on CD138 expression. Positivity for DENV infection was determined for each B cell subset based on expression of viral protein NS3.

Supplementary Figure 5 Representative gating strategy used to assess activation markers CD69, CD86 and proliferation marker Ki-67 in B cells isolated from dengue patients. B cells from dengue patients were stained for CD20 and CD27 to determine naive B cells (CD20^+^CD27^−^) and memory B cells (CD20^+^CD27^+^). Uninfected and DENV infected cells were defined as NS3^−^ and NS3^+^ based on expression of DENV NS3. NS3^−^ and NS3^+^ cells were further gated for CD69, CD86, and Ki-67 to assess activation and proliferation of B cells.

Supplementary Figure 6 Representative gating strategy for *in vitro* plasmablast and plasma cell development after DENV infection in B cells. Total cells were gated followed by exclusion of doublets and dead cells. CD20^+^ B cells were gated and further sub-gated based on expression of CD27. CD20^+^CD27^+^ B cells were defined as plasmablasts (CD20^+^CD27^+^CD38^+^CD138^−^) and plasma cells (CD20^+^CD27^+^CD38^+^CD138^+^).
