# Supplementary figures and images for: Direct Infection of B Cells by Dengue Virus Modulates B Cell Responses in a Cambodian Pediatric Cohort
Source: Front Immunol. 2021 Feb 12;11:594813. doi: 10.3389/fimmu.2020.594813 (PMC7907177; doi:10.3389/fimmu.2020.594813)

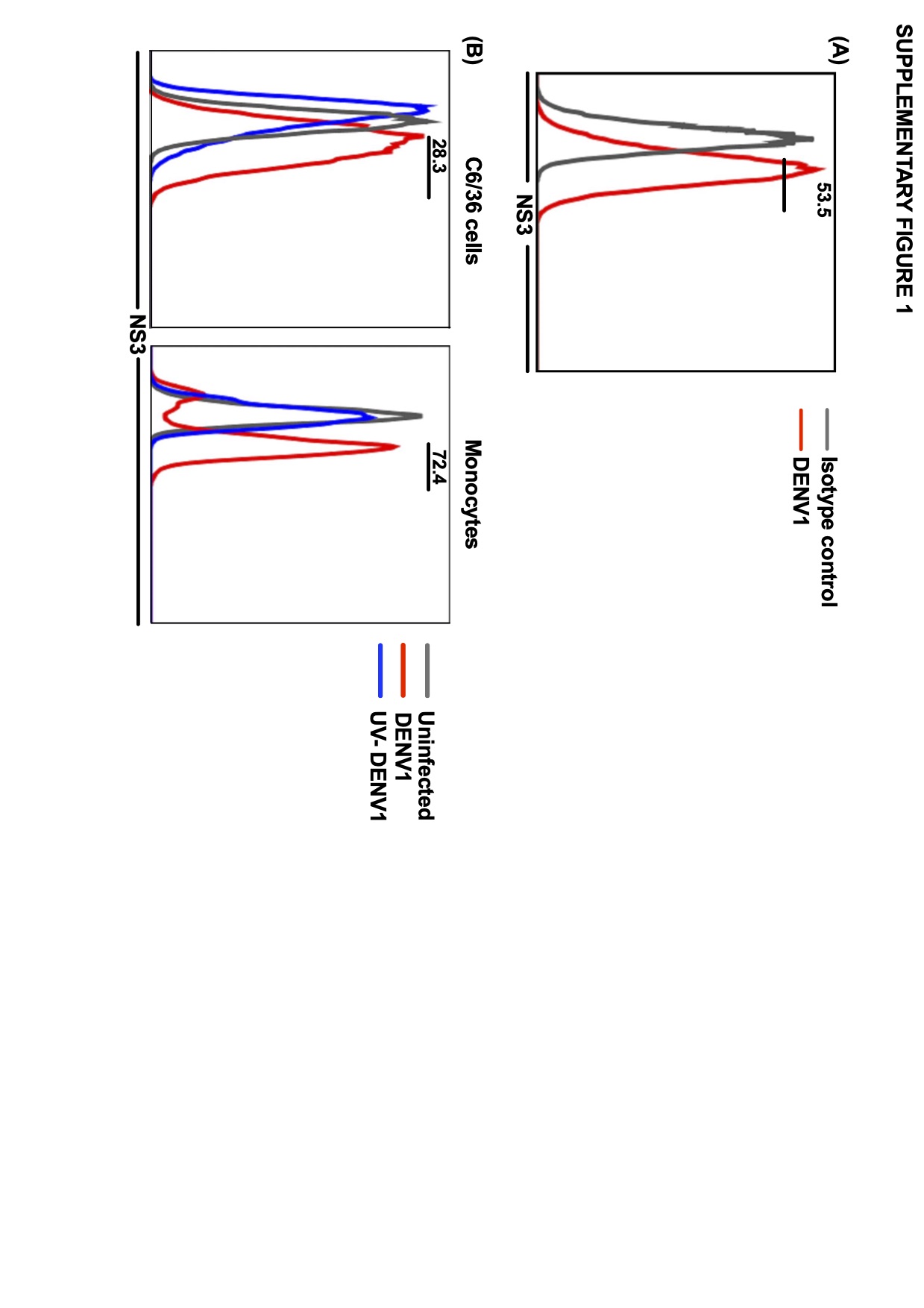

Supplement: Supplementary Figure 1 — Optimization of anti-DENV NS3 antibody. (A) C6/36 cells were infected with DENV-1 at MOI of 20 for 24 h and stained with rabbit polyclonal anti-DENV NS3 antibody and secondary goat antibody conjugated with AF488. A non-specific rabbit polyclonal antibody was used as negative control. (B) C6/36 cells and CD14+ monocytes isolated from healthy donors were infected in vitro with DENV1 or UV-inactivated DENV1 (UV-DENV1) at MOI 10 and stained at 24 h post infection with rabbit polyclonal anti-DENV NS3 antibody and secondary goat antibody conjugated with AF488. [file Image_1.jpeg]

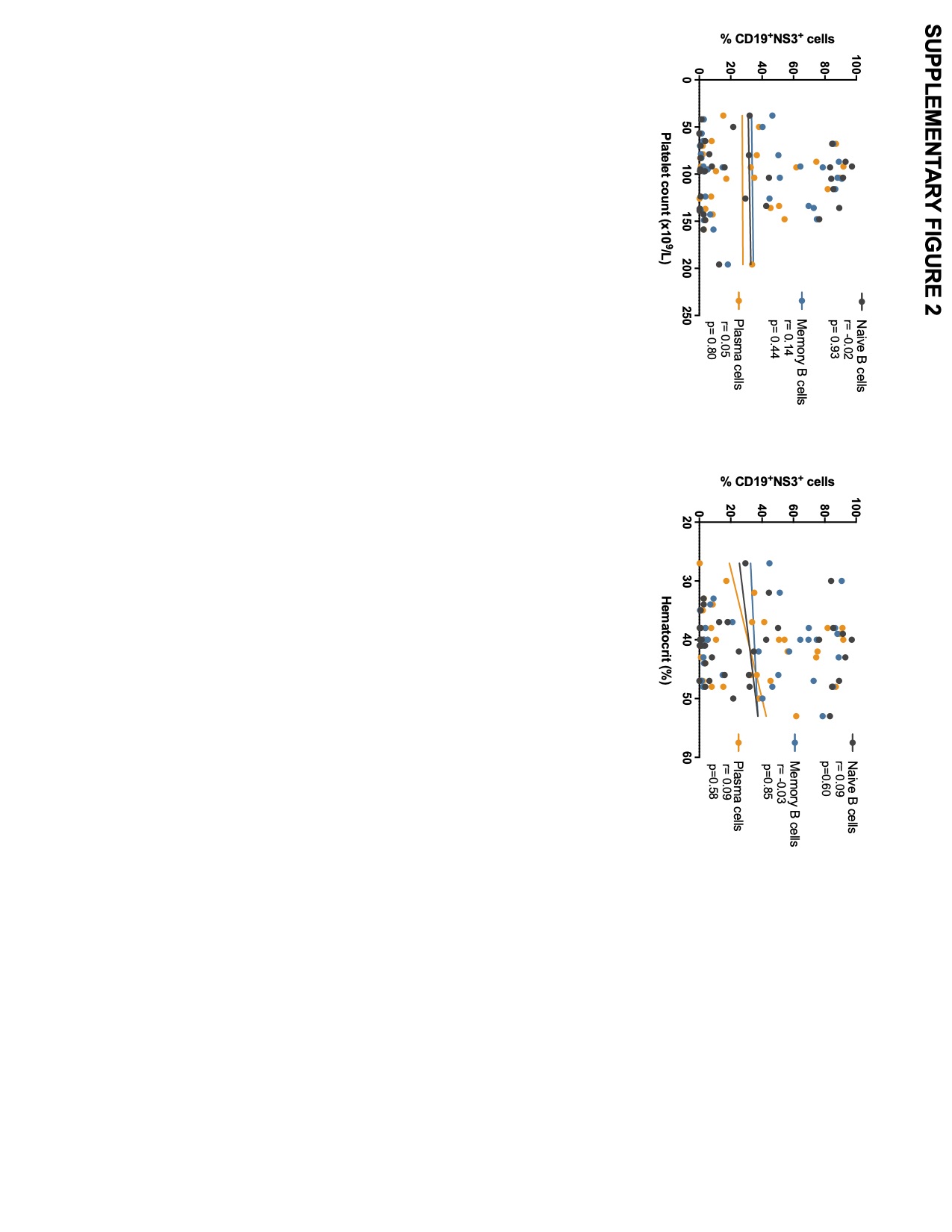

Supplement: Supplementary Figure 2 — No correlation between biological parameters and percentages of DENV-infected B cells. Correlations between platelet counts and hematocrit levels at hospital admittance and percentages of DENV-infected B cell subsets were determined by Spearman’s correlation. [file Image_2.jpeg]

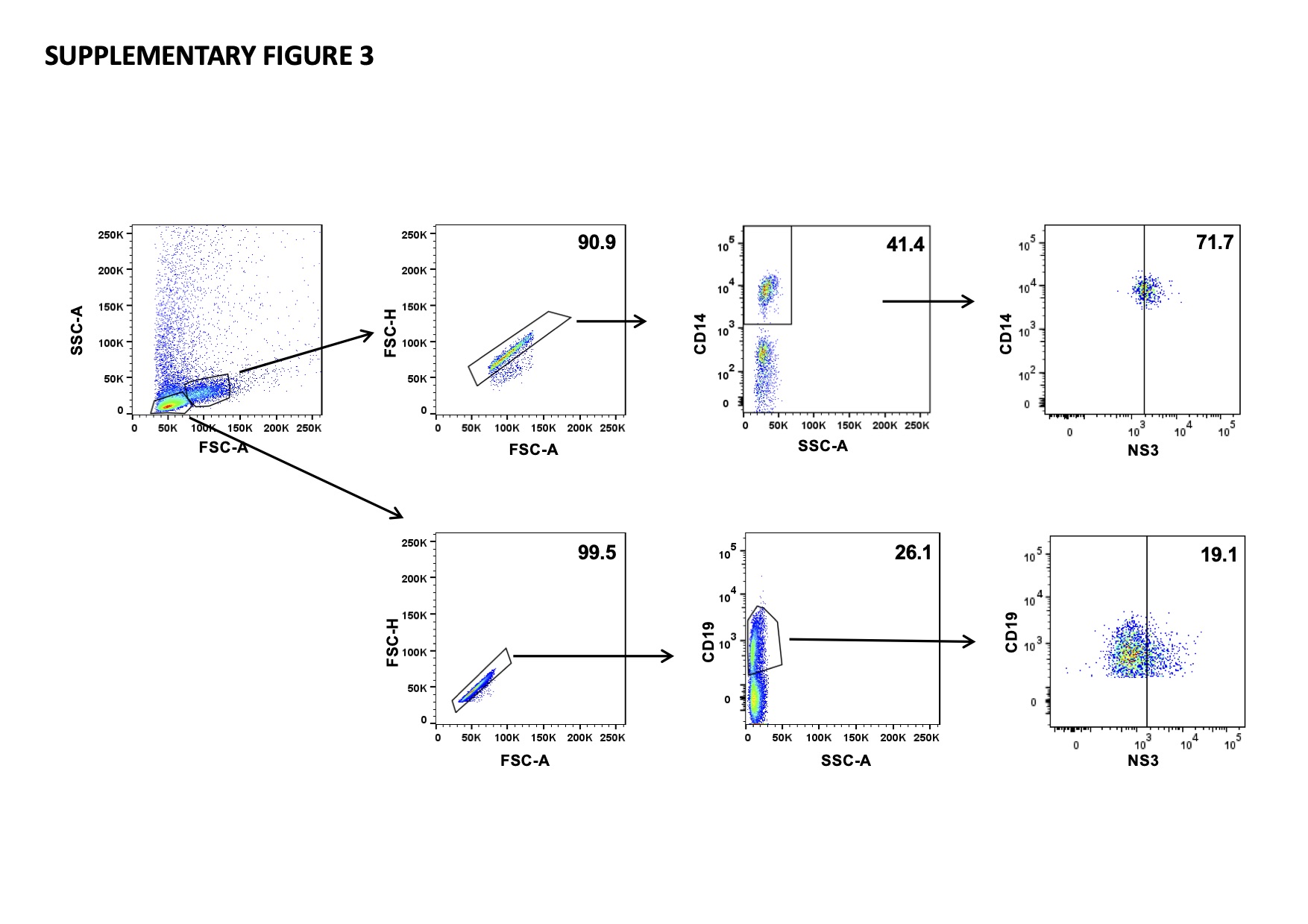

Supplement: Supplementary Figure 3 — Representative gating strategy used to detect DENV infection in B cells and monocytes in PBMCs from dengue patients. PBMCs were gated for lymphocytes and monocytes followed by removal of doublets. Single CD14+ monocytes and CD19+ B cells were gated and DENV NS3+ cells were selected. [file Image_3.jpeg]

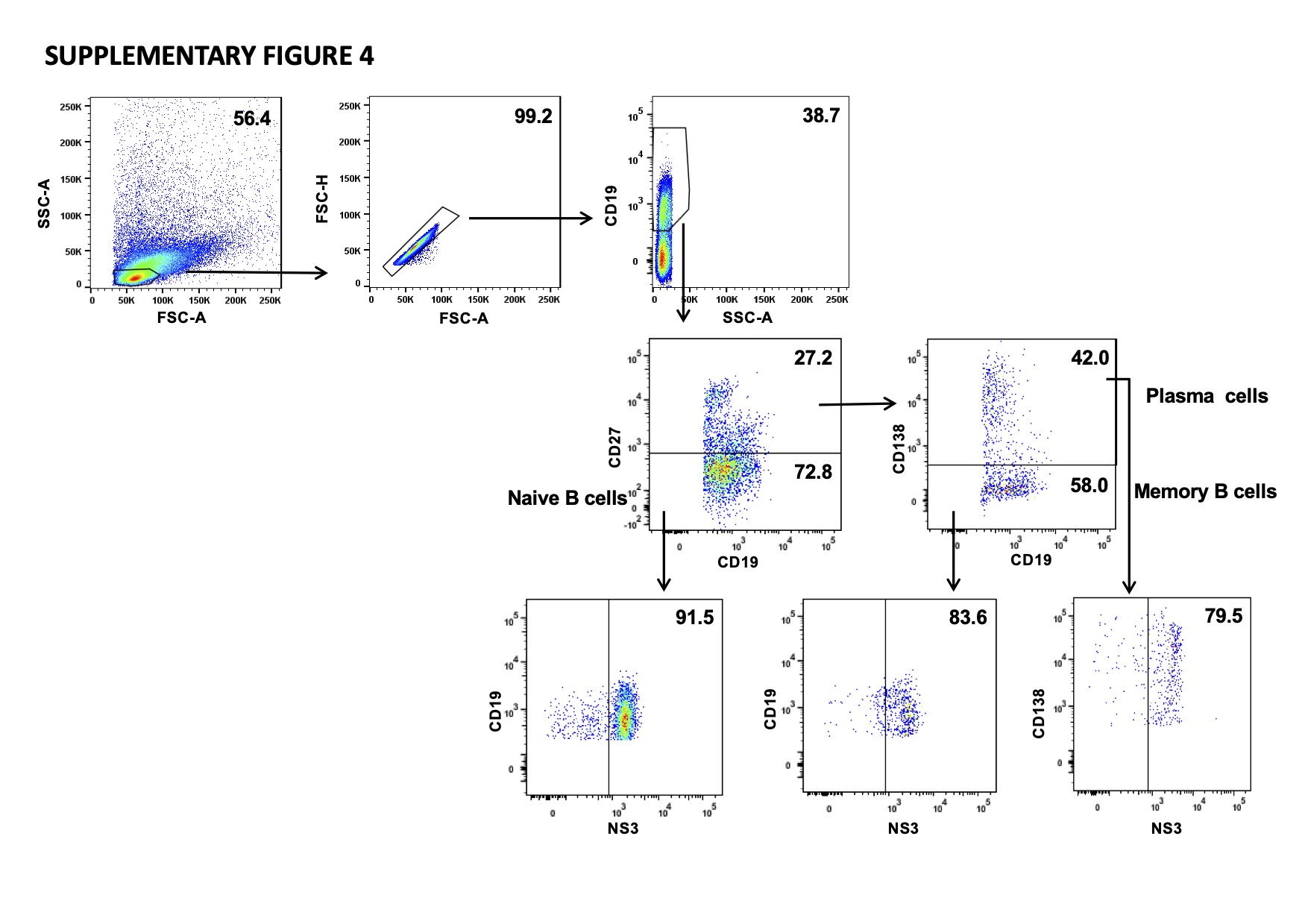

Supplement: Supplementary Figure 4 — Representative gating strategy used to detect DENV infection in B cell subsets in PBMCs from dengue patients. Lymphocytes from PBMCs were further gated for CD19+ B cells. Based on expression of CD19 and CD27, total B cells were further gated as naïve B cells (CD19+CD27−). CD27+ B cells were gated as memory B cells (CD19+CD27+CD138−) and antibody secreting cells (CD19+CD27+CD138+) based on CD138 expression. Positivity for DENV infection was determined for each B cell subset based on expression of viral protein NS3. [file Image_4.jpeg]

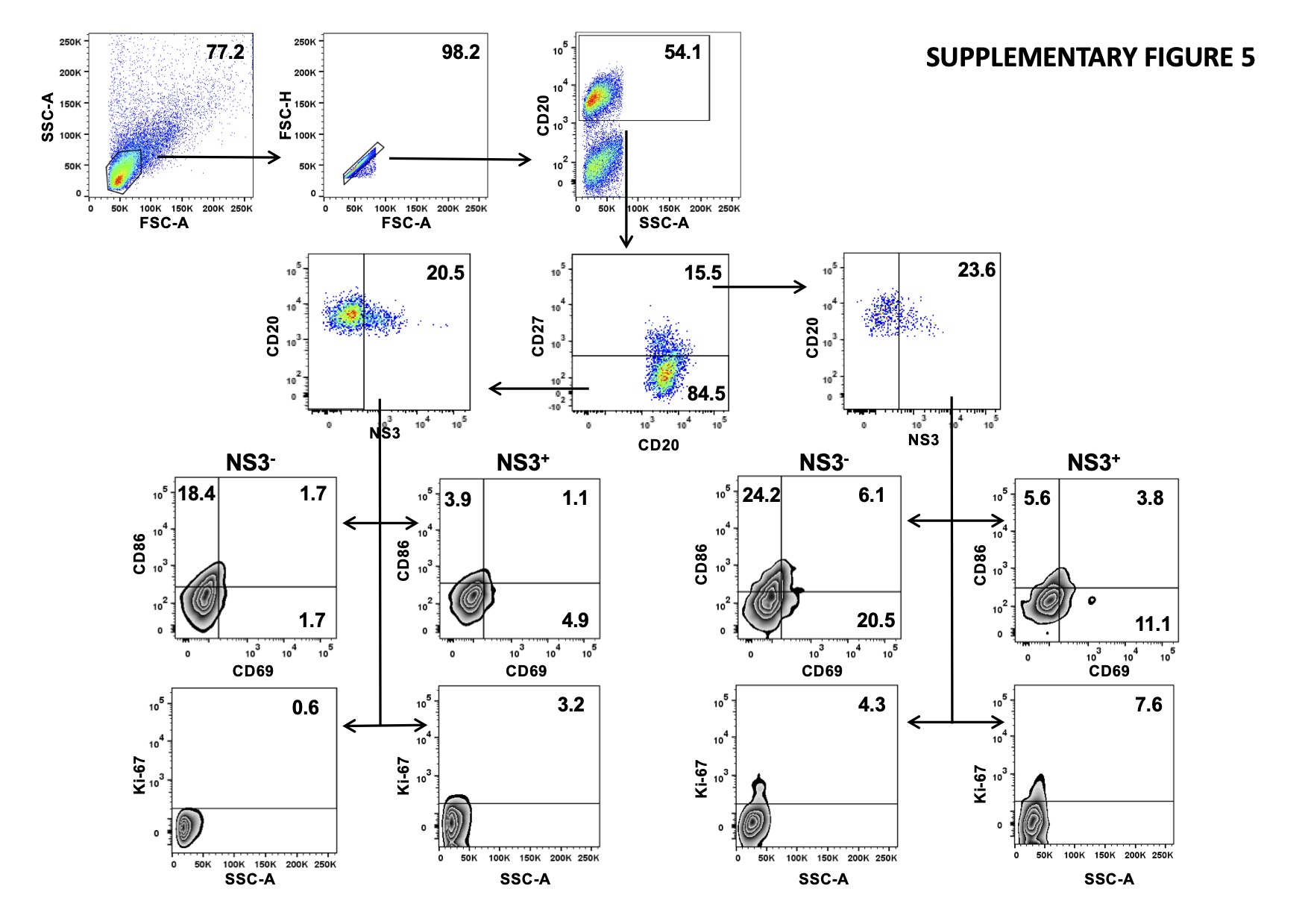

Supplement: Supplementary Figure 5 — Representative gating strategy used to assess activation markers CD69, CD86 and proliferation marker Ki-67 in B cells isolated from dengue patients. B cells from dengue patients were stained for CD20 and CD27 to determine naive B cells (CD20+CD27−) and memory B cells (CD20+CD27+). Uninfected and DENV infected cells were defined as NS3− and NS3+ based on expression of DENV NS3. NS3− and NS3+ cells were further gated for CD69, CD86, and Ki-67 to assess activation and proliferation of B cells. [file Image_5.jpeg]

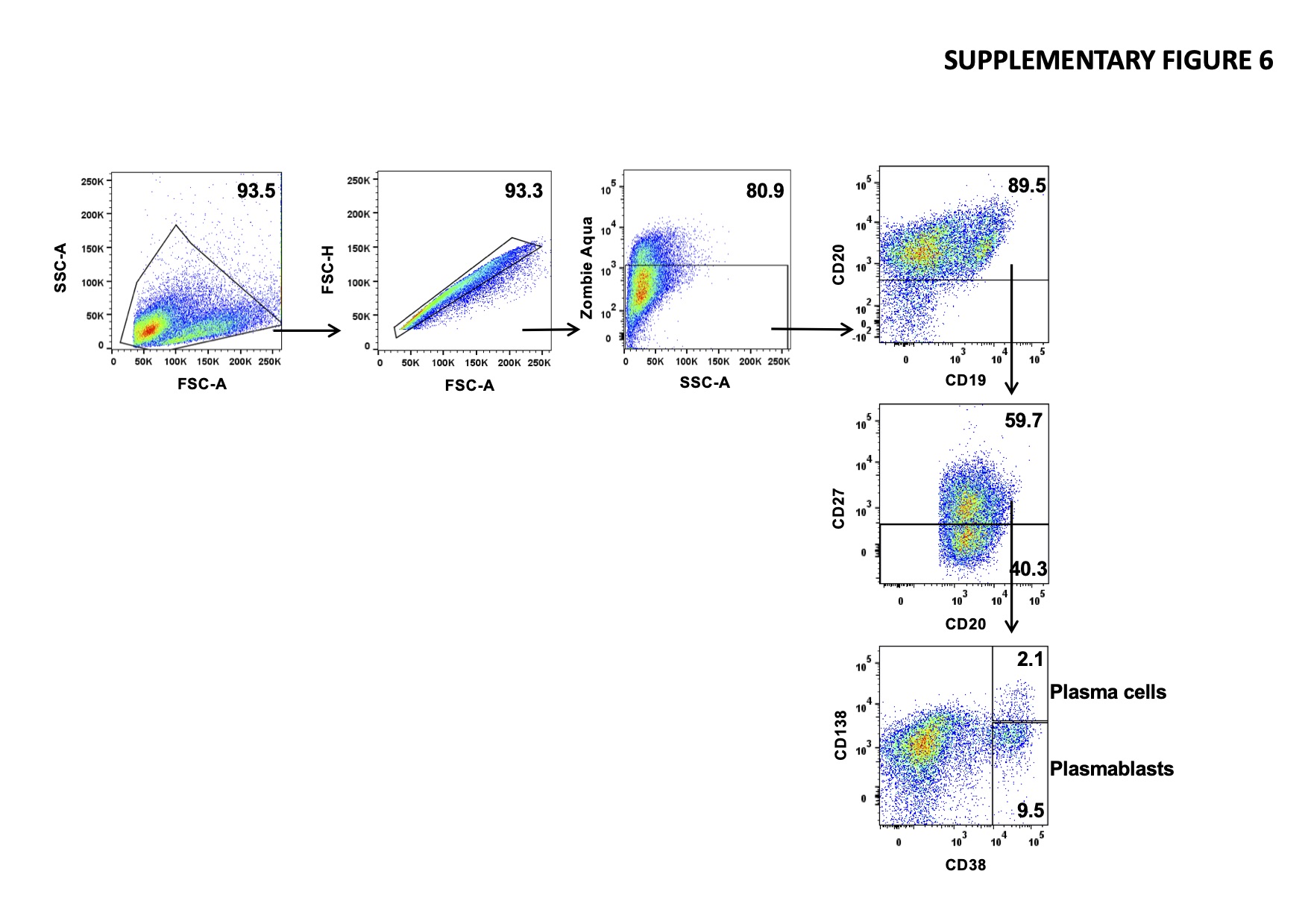

Supplement: Supplementary Figure 6 — Representative gating strategy for in vitro plasmablast and plasma cell development after DENV infection in B cells. Total cells were gated followed by exclusion of doublets and dead cells. CD20+ B cells were gated and further sub-gated based on expression of CD27. CD20+CD27+ B cells were defined as plasmablasts (CD20+CD27+CD38+CD138−) and plasma cells (CD20+CD27+CD38+CD138+). [file Image_6.jpeg]
